# Scene management approaches in dealing with drowning injuries: a review study 

**Published:** 2022-01

**Authors:** Maryam Lari Siahkal, Mohammad Reza Shiri, Tahereh Zahedsefat, Alireza Mohsenipour Foumani, Leila Shoaei Deilami

**Affiliations:** ^*a*^ BSc in Nursing, Health Education Expert of Siahkal Health Center, Guilan University of Medical Sciences, Rasht, Iran.; ^*b*^ Pediatrician, Manager of Siahkal Health Center, Guilan University of Medical Sciences, Rasht, Iran.; ^*c*^ BSc in Nursing, Master of Business Administration, Manager of Guilan Nursing Services, Guilan University of Medical Sciences, Rasht, Iran.; ^*d*^ BSc in Nursing, MA in Electronic Learning Planning, Social Security Organization, Tehran, Iran.; ^*e*^ BSc in Nursing, Supervisor of Amir Al-Momenin Hospital, Guilan University of Medical Sciences and Health Services, Rasht, Iran.

## Abstract

**Background:**

Although drowning patients need emergency medical care, the majority of patients are never brought to treatment centers. Most drowning accidents occur to children under one, adolescents and young people, and pre-school boys in bathtubs, ponds, lakes, rivers, seas, and swimming pools, respectively. Rescue operations are critical and must start immediately. Death in drowned people is due to laryngospasm and lung damage, resulting in hypoxemia and affecting the brain and other body systems.

**Methods:**

In the present review study, databases and scientific engines such as Google Scholar were searched using various keywords including “rescue”, “drowning”, and “management” from 2000 to 2020.

**Results:**

The results show that improving rescue equipment quantitatively and qualitatively in coastal provinces is essential. Therefore, basic measures are critical for enhancing personal and public health. Proper attention and management of some fundamental rescue issues can prevent tragic accidents. Water immersion is traumatic for the injured person leading to ventilation lack due to prolonged hypoxemia. The injured person suffers from apnea and laryngospasm of varying severity and the duration of hypoxemia, and acidosis develops in the body due to initial shortness of breath, which results in cardiac arrest and ischemia of the central nervous system. After drowning in the first minute, laryngeal spasms due to aspiration and vagal stimulation occur. Spasm disappears and fluid aspiration with a larger volume appears in the third minute. During three to six minutes, blood circulation is disrupted, acidosis occurs and brain damage begins. After six minutes, anoxia and tissue damage are observed.

**Conclusions:**

Various life-threatening criteria have to be evaluated to deal with an injured person immediately, including immersion time, water temperature, head, neck, and spine injuries, surviving ways, response to resuscitation, and essential factors and symptoms such as persistent cough, dyspnea, or apnea, change in the level of consciousness, vomiting, consumption of alcohol or drugs and underlying diseases.

**Keywords:**

Rescue, Drowning, Management

